# Editorial: Edible halophytes for a sustainable agriculture: from neglected species to new crops

**DOI:** 10.3389/fpls.2024.1504271

**Published:** 2024-10-22

**Authors:** Angelo Signore, Massimiliano Renna, Juan A. Fernández

**Affiliations:** ^1^ Department of Soil, Plants and Food Sciences, University of Bari Aldo Moro, Bari, Italy; ^2^ Department of Agronomical Engineering, Technical University of Cartagena, Cartagena, Spain

**Keywords:** halophytes, sustainable, neglected, new crops, edible

Soil salinity is one of the most significant challenges in agriculture, particularly among abiotic stresses. Approximately 10% of the world’s arable land is affected by salinity or sodium, with 25% to 30% of irrigated land suffering from salinity, rendering much of it commercially unproductive. However, halophytes can thrive in saline environments, even at concentrations exceeding 200 mM NaCl. These species are gaining attention in the food industry for two key reasons: (i) their productivity in challenging conditions, such as high salinity and low water availability, often surpasses that of traditional crops, and (ii) their rich nutritional content—including phenolics, proteins, lipids, and essential minerals like potassium, calcium, and magnesium, as well as other bioactive compounds—warrants further exploration. Additionally, many halophytes are classified as neglected or underutilized species (NUS). Incorporating these species into cropping systems could enhance the sustainability of food production by promoting biodiversity, improving climate adaptability, and reducing environmental impacts.

The Research Topic, titled “*Edible Halophytes for Sustainable Agriculture: From Neglected Species to New Crops*,” aimed to identify edible halophyte species for cultivation and promotion as new crops. The goal was to transition these species from their “neglected and underutilized” status to active cultivation, broadening the variety of plants used for human nutrition, particularly in arid, semi-arid, and marginal regions in response to climate change.

Xu et al. examined the impact of water-nitrogen interactions on the water-salt environment and root distribution in the root zone of *Suaeda salsa*. They employed a full factorial design with three irrigation levels and three nitrogen levels. The authors found that, under the same irrigation level, the distribution trend of soil substrate suction was consistent across different nitrogen application rates. As both irrigation volume and nitrogen application rates increased, the total root weight density of the saline alkali fluffy root system also increased, with the root distribution shifting from a “narrow deep type” to a “wide shallow type.” At the same nitrogen application rate, the biomass, ash content, and salt uptake of *S. salsa* in saline soil initially increased and then decreased with rising irrigation volume, peaking at the intermediate irrigation level. In conclusion, intermediate levels of both irrigation and nitrogen application are likely the most effective for drip irrigation of *S. salsa* in extremely arid regions.

Conversa et al. investigated two distinct leaf morphotypes (entire lamina and pinnatifid lamina) of sea rocket (*Cakile maritima* subsp. *maritima* Scop.) as high-nutritional food. They measured various bio-morphological traits, main inorganic ions, key antioxidants, antioxidant activity, photosynthetic gas exchange, and chlorophyll fluorescence. The morphotype with pinnatifid lamina exhibited greater accumulation of photo-protective pigments, higher photosynthetic activity, increased transpiration rates, and greater stomatal conductance, alongside reduced non-photochemical quenching. In contrast, the morphotype with entire lamina showed higher concentrations of cations and vitamin C. No significant differences were observed between the morphotypes in terms of phenolic concentrations, flavonoids, or glucosinolates. Although the pinnatifid lamina morphotype appears better adapted to the water and nutrient scarcity typical of southern Italy, both morphotypes hold potential as high-nutritional foods.

Gu et al. conducted research on the salt tolerance mechanisms of *Glaux maritima* through phenotypic, physiological, and transcriptomic analyses. After exposure to high salt stress, the leaf cells of *G. maritima* exhibited a decrease in volume and became densely arranged. Physiologically, the maximum salt tolerance threshold for *G. maritima* leaves was found to be 600 mM/L. At this concentration, proline content, relative conductivity, and the activities of superoxide dismutase (SOD), peroxidase (POD), and catalase (CAT) reached their peak levels. Transcriptome data from three experimental groups were analyzed, revealing six essential genes related to proline synthesis and five important genes associated with SOD and CAT enzyme activities. Additionally, two genes involved in CAT enzyme activity were identified as playing a significant role in the MAPK signaling pathway. Trend analysis indicated that MAPK signaling regulation, phytohormone regulation, glutathione metabolism, and flavonoid biosynthesis pathways were crucial for regulating the salt tolerance of *G. maritima*.

Finally, Gimenéz et al. applied a cascade cropping system to cultivate *Salicornia fruticosa*, aiming to reduce nutrient discharge and assess the impact of four concentrations of exogenous melatonin on the growth and quality of this halophyte species. The authors found that melatonin application increased yield and maximized water use efficiency, particularly when plants were grown in compost leachate. The highest nitrogen use efficiency was observed in plants grown in peat leachate. Overall, shoots cultivated in peat leachate exhibited the best phytochemical profile. These findings suggest that *S. fruticosa* can be effectively grown using leachate from a previous crop in a floating system, and that exogenous melatonin application enhances both the yield and nutritional quality of *Salicornia* shoots.

In summary, this Research Topic brings together various research efforts to highlight recent progress in the cultivation and physiology of edible halophytes for sustainable agriculture. These studies are essential to assess their potential as new vegetable crops for marginal areas and intensive cropping systems, with applications in the production of innovative plant products.

